# Effects of diacerein on cartilage and subchondral bone in early stages of osteoarthritis in a rabbit model

**DOI:** 10.1186/s12917-015-0458-x

**Published:** 2015-07-02

**Authors:** María Permuy, David Guede, Mónica López-Peña, Fernando Muñoz, Jose-Ramón Caeiro, Antonio González-Cantalapiedra

**Affiliations:** Veterinary Clinical Sciences, University of Santiago de Compostela (USC), Campus Universitario, s/n, 27002, Lugo, Spain; Trabeculae S.L., Parque Tecnolóxico de Galicia, 32900 San Cibrao das Viñas, Ourense, Spain; Orthopedic Surgery Service, USC University Hospital Complex, Travesía de Choupana, s/n, 15706, Santiago de Compostela, Spain

**Keywords:** Bone, Cartilage, Diacerein, Osteoarthritis, Rabbit model, Synovial membrane

## Abstract

**Background:**

Osteoarthritis is thought to be the most prevalent chronic and disabling joint disease in animals and humans. At present, there is no ideal treatment option. The aim of this study was to assess the effects of the treatment with oral diacerein on articular cartilage, synovial membrane and subchondral bone in an experimental rabbit model of osteoarthritis by micro-CT evaluation and histological analysis. To this purpose, osteoarthritis was surgically induced on one knee of 16 rabbits using the contralateral knee as healthy controls. Treatment was started three weeks later and lasted eight weeks. Animals were divided into two groups for treatment: Placebo (treated daily with oral saline) and diacerein (treated orally with 1.5 mg/kg/day of diacerein).

**Results:**

Sample analysis revealed that this model induced osteoarthritis in the operated knee joint. Osteoarthritis placebo group showed a significant increase in non-calcified cartilage thickness and volume with respect to the control placebo group and important changes in the synovial membrane; whereas the parameters measured in subchondral bone remained unchanged. In the osteoarthritis diacerein-treated group the results showed an improvement with respect to the OA placebo group in all parameters, although the results were not statistically significant.

**Conclusion:**

The results of this animal study suggested that the diacerein treatment for OA may be able to ameliorate the swelling and surface alterations of the cartilage and exert an anti-inflammatory effect on the synovial membrane, which might contribute to OA improvement, as well as an anabolic effect on subchondral trabecular bone.

## Background

Osteoarthritis (OA) is a heterogeneous chronic disease affecting all tissues of the synovial joint. It is now accepted that the disease etiology is multiple and involves various mechanical, biochemical and genetic factors, as well as molecular and enzymatic feedback loops. Osteoarthritic changes are primarily the result of a disturbance in the remodelling processes of tissues resulting from the failure of cells to maintain a homeostatic balance; as the disease advances, the catabolic process exceeds the anabolic ones leading to progressive joint tissue lesions. The morphological changes observed in OA include a variable degree of synovial inflammation, principally at the clinical stage of the disease, which in turn produces inflammatory mediators (inflammatory cytokines), which play a pivotal role in the pathophysiological mechanisms of the OA [1, 2].

The pharmacological treatment of OA includes different types of drugs that can be classified on basis of their mode of action. Current treatment options are focused on pain relief and improvement of joint function, including analgesics and non-steroidal anti-inflammatory drugs; unfortunately, their use in chronic treatments has been limited by the deleterious side effects on cartilage [3] and gastrointestinal tract [4]. Recent studies have described drugs with the ability of providing symptomatic relief by targeting the underlying pathology of OA, particularly in cartilage and subchondral bone, and with less secondary effects; such agents have been classified as symptomatic slow-acting drugs for OA (SYSADOAs) which are expected to delay, stabilise or reverse the pathological changes in OA joints, limiting the progression of the disease [5]. This group of drugs includes diacerein, an anthraquinone derivative (4,5-diacetyloxy-9,10-dioxo-anthracene-2-carboxylic acid), which is an oral anti-inflammatory, analgesic and antipyretic agent developed specifically for the treatment of OA.

It is actually well-known the contribution of pro-inflammatory cytokines to cartilage degradation in OA [6, 7] including interleukin-1 (IL-1) which stimulates the degradation process of cartilage and suppresses cartilage matrix synthesis. Rhein, the active metabolite of diacerein, has shown *in vitro* and *in vivo* the inhibition of the IL-1β, as well as the reduction of the collagenase production by joint chondrocytes, the reduction of fibrinolytic activity in synovial fluid [8, 9] and the stimulation of the production of cartilage growth factors [10]. This way of action turned diacerein into an interesting option for the treatment of OA without the side effects of NSAIDs [11].

In a previous study in dogs [12], diacerein treatment significantly reduced the severity of morphologic changes in the knee joint compared to placebo, using the SFA (Societé Française d’Arthroscopie) score and, in the same study, the Mankin scores were lower for the diacerein group. In another study in rats [13], the treatment with diacerein produced the best functional results represented by a greater joint extension. In addition, several trials in humans suggested beneficial role of diacerein in hip and knee OA with results similar to those obtained by NSAIDs during the active treatment phase [14]. Bartels *et al*. (2010) [15] described a small reduction of pain in patients treated with diacerein during the first 6 months, whereas Pavelka *et al*. (2007) [16] reported a statistical superiority of diacerein versus placebo in pain control and tenderness of the joint; Singh *et al*. (2012) [17] reported an additive effect with diclofenac for pain and improvement of the joint motion.

The aim of this study was to test the effectiveness of the treatment with oral diacerein in an experimental early rabbit model of OA; histology and histomorphometry techniques were used and the results were compared with a new methodology to determine the cartilage and subchondral bone structure, using micro-computed tomography (micro-CT) without contrast techniques.

## Methods

### Pre- and postoperative care. Osteoarthritis induction

Sixteen adult female (6–7 months old, mean weight 5 Kg) healthy New Zealand white rabbits (Granja San Bernardo, Navarra, Spain) were used, after approval of the protocol by the Ethical Committee of the University of Santiago de Compostela (Reference number: AE-LU-002/09/FUN01/PAT.(06)E). All animals were subjected to surgery and throughout the procedures they were housed in cages at the Animal Research Facility, University of Santiago de Compostela, Lugo, Spain. The procedures were performed according to local and European regulations on care and use of research animals, and this paper was written in accordance with the ARRIVE statement [18]. The rabbits were monitored daily during the entire experimental procedure by an accredited veterinarian, trained in laboratory animal science.

The protocol used has been deeply described in a recently published article [19]. Summarising, OA was induced by anterior cruciate ligament transection (ACLT) and partial medial meniscectomy of one knee (randomly chosen) using the contralateral knee as untreated control. Animals were anaesthetised using isoflurane (2.5–4 %, Isova-vet, Schering-Plough, Madrid, Spain) after premedication with a combination of medetomidine (50 μg/Kg IM; Domtor, Esteve, Barcelona, Spain) and ketamine (25 mg/Kg IM; Imalgène 1000, Merial, Toulouse, France). Each animal received peri- and post-operative buprenorphine (1 mg/Kg IM; Buprex, RB Pharmaceuticals, Berkshire, UK), antibiotic prophylaxis with enrofloxacin for a week (15 mg/Kg SC once a day; Ganadexil 5 %, Invesa, Barcelona, Spain) and meloxicam (20 μg/Kg SC; Metacam, Boehringer Ingelheim España, Barcelona, Spain) for three days after surgery.

**Figure 1:**
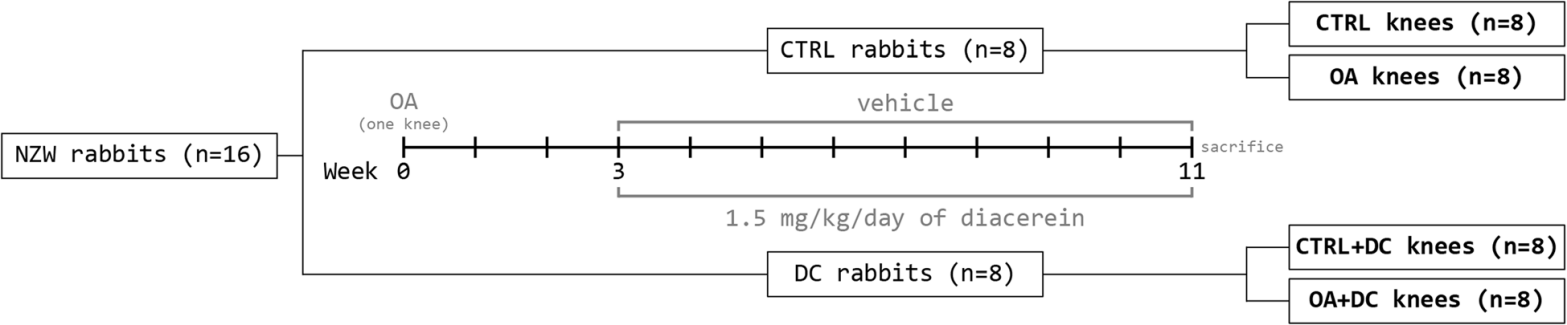
Experimental design. Groups of treatment

Three weeks after surgery, eight animals were randomly selected in each experimental group. The control group was treated with 5 mL of saline (NaCl 0.9 %) and the treatment group with 1.5 mg/Kg of diacerein. Both treatments were administered daily and directly into the mouth using a 5 ml syringe. The group distribution is represented in Fig. 1.

### Necropsy and preparation of histological specimens

Rabbits were sacrificed by sodium pentobarbital overdose (100 mg/Kg IV; Dolethal, Vétoquinol, France) after sedation with ketamine (25 mg/Kg IM; Imalgène 1000, Merial, Toulouse, France). A complete necropsy of all the animals was performed to evaluate the presence of any alterations other than those in knee joints. After dissecting the articulation, two cylinders of 2.9 mm diameter and 8 mm length were obtained from the medial femoral condyle at the same anatomical locations of both knees of every animal and, within the same procedure a section of the articular capsule adjacent to the patellar ligament was obtained.

For evaluation, samples were divided in four groups (Fig. 1): CTRL: healthy non-operated knees of the placebo-treated animals (*n* = 8); OA: operated knees of the placebo group (*n* = 8); CTRL + DC: non-operated healthy knees of the diacerein-treated animals (*n* = 8); OA + DC: osteoarthritic knees of the diacerein-treated animals (*n* = 8).

One trephine was decalcified with EDTA (ethylenediaminetetraacetic acid) in acid buffer (Osteodec, Bio-Optica, Milano, Italy) and, together with the articular capsule, was paraffin-embedded, sectioned and stained with haematoxylin-eosin (H-E) and safranin O-fast green. The synovial membrane slides were stained with H-E. The second trephine-cylinder was firstly used to evaluate the architecture of the cartilage and subchondral bone by micro-CT and later it was processed for undecalcified ground sections according to the method described by Donath (1985) [20], and stained using the Lévai-Laczkó method [21].

#### Microscopic evaluation

The gradation of the samples was performed in the same way as in a previously published article [19]

The synovial membrane and the decalcified bone and cartilage core biopsies were evaluated by two independent observers according to the already published guidelines [22, 23]. The gradation of damage to the structures was as follows: in cartilage, chondrocyte and proteoglycan pathology from 0 (normal) to 4 (completely affected) and in tidemark and synovial membrane from 0 (normal) to 2 (osteoarthritic). In cartilage samples the parameters evaluated were: the cartilage pathology (cartilage surface characteristics), chondrocyte pathology (chondrocyte density and distribution, paying special attention to the clusters formation), proteoglycan pathology (degree of staining), tidemark integrity. In synovial membrane slides: lining cell characteristics (number of superficial cell layers), hyperplasia (presence or absence and its characteristics), and the presence or absence of cellular infiltrations.

**Figure 2:**
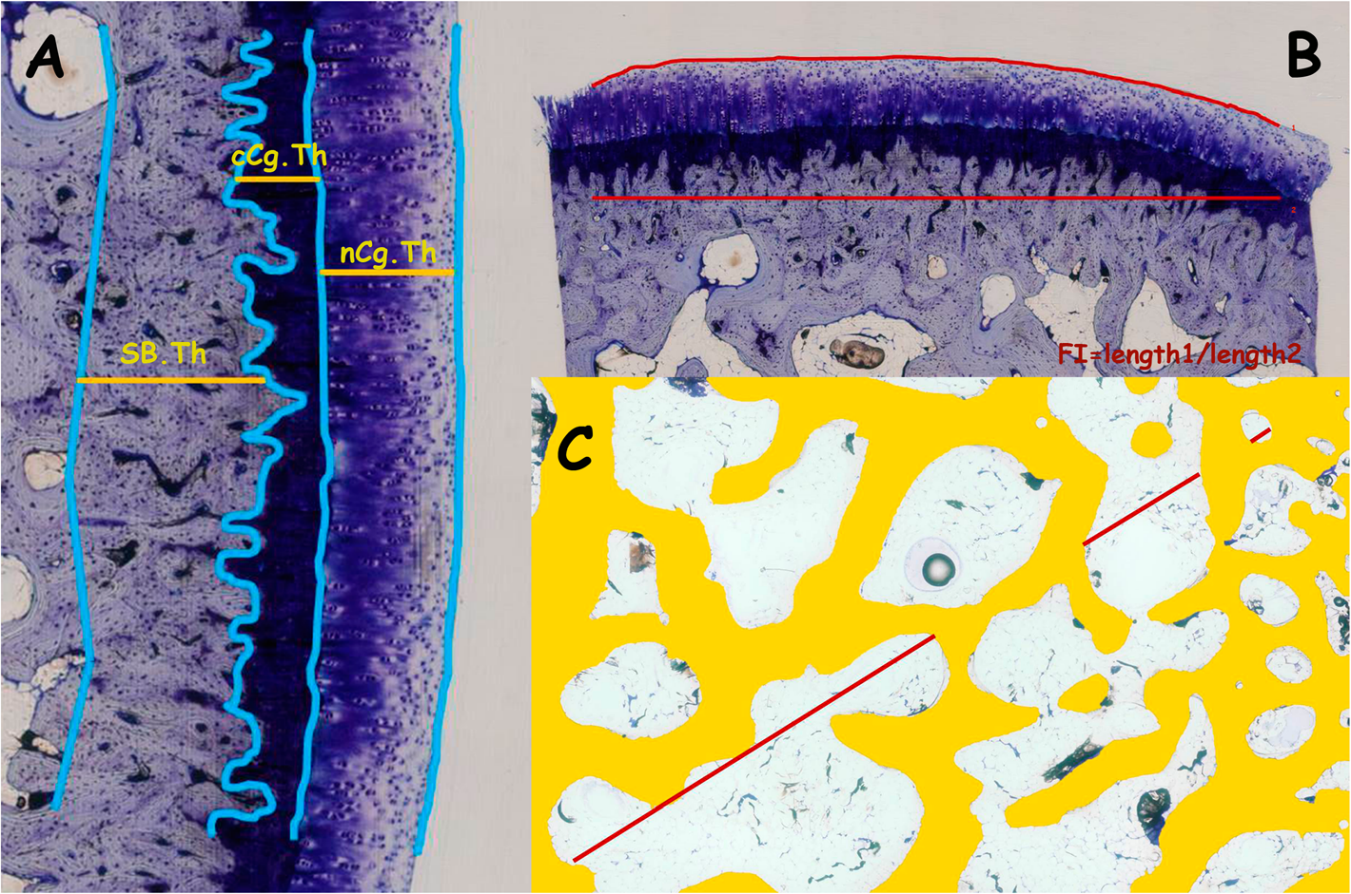
Representative images of the measurements made in undecalcified sections: **a** cartilage and subcondral bone cortical thickness (nCg.Th; cCg.Th; Cg.Th = nCg.Th + cCg.Th) SB.Th). **b**. Surface undulation (FI). **c** Trabecular subchondral bone measurements in a ROI. Tb.A: % of trabecular bone in the ROI; Tb.Sp measured on the diagonal of ROI (Tb.Sp = (1/Tb.N)-Tb.Th)

Quantitative histology was performed using undecalcified sections applying previously published [24] morphological parameters by a masked examiner using PC-based image analysis programmes: Cell-sens 1.5 (Olympus Corporation, Japan) and Micro-image 4.0 (Media cybernetics, Bethesda, MD, USA). Firstly the reference lines were identified (Fig. 2) and after that, the programme automatically calculated the mean distances between these lines.

The evaluated parameters were [19]: Subchondral bone cortical thickness (SB.Th), total cartilage thickness (Cg.Th) and separately non-calcified (nCg.Th) and calcified cartilage thickness (cCg.Th) using the tidemark as reference (Fig. 2a), cartilage surface undulations (FI) (Fig. 2b), trabecular subchondral bone area (Tb.A) and trabecular separation (Tb.Sp). Tb.A and Tb.Sp were measured in a single subchondral bone region of interest (ROI) of 4x2mm beginning immediately after the subchondral cortical bone and including the entire width of the core biopsy except the margins (Fig. 2c). Tb.A was defined as the percentage of trabecular bone in this region and Tb.Sp as the mean distance between trabeculae.

### Micro-computed tomography (micro-CT)

Undecalcified sections were evaluated with a high-resolution micro-CT (SkyScan 1172, Bruker micro CT NV, Kontich, Belgium). Two scans were performed for each sample, one to assess the microstructure of the subchondral bone and the other to visualise the articular cartilage using previously published parameters [19]. Images were reconstructed based on Feldkamp’s convolution back-projection algorithm [25], and segmented into binary images. The following direct metric indices were measured: bone volumetric fraction (BV/TV), trabecular thickness (Tb.Th) and separation (Tb.Sp), and trabecular number (Tb.N) [26]. Directly assessed non-metric parameters were also calculated, including the trabecular bone pattern factor (Tb.Pf), which is an inverse measure of trabecular connectivity [27], the structural model index (SMI), which estimates the prevalence of rod-like or plate-like trabeculae [28], and the degree of anisotropy (DA) describing the orientation of trabeculae [29]. Additionally, the volumetric bone mineral density (vBMD) was determined by direct calibration against attenuation coefficients of two hydroxyapatite phantoms (250 and 750 mg/cm^3^). Regarding the cartilage, the average thickness of the non-calcified cartilage layer (nCg.Th) and its volume (nCg.V) were 3-D quantified.

### Statistical analysis

The statistical analysis was performed with SPSS 19 for Windows (IBM, Armonk, NY, USA). The normality of the variables was checked by the Shapiro-Wilk test. The statistical comparison of normal variables was performed using ANOVA and the Levene’s test was used to assess the equality of variances. *Post-hoc* analysis was carried out using the Tukey’s HSD test for parameters with equal variances or the Games-Howell test for parameters with different variances. For non-normal variables, a statistical comparison was performed using the Kruskal-Wallis H test and a *post-hoc* analysis using the Dunn’s test. The statistical significance level was set at *p* < 0.05 for all parameters.

## Results

No changes in weight or general condition were observed during the experimental protocol. The only outstanding fact was that animals treated with diacerein presented urine discoloration; however, no pathological changes in the kidneys or the rest of the organs were detected in necropsies.

Out of the total of 32 harvested joints, except for one excluded due to infection, 31 presented an adequate status to be histologically analysed.

### Quantitative histological results

Mean and standard deviation (SD) of the parameters quantified on undecalcified samples are shown in Table 1.

**Table 1:** Quantitative histological results

	CTRL	OA	CTRL + DC	OA + DC
	mean ± std.dev	mean ± std.dev	mean ± std.dev	mean ± std.dev
Subchondral bone				
Tb.A (mm^2^)	46.37 ± 5.41	47.93 ± 7.01	50.69 ± 7.34	45.54 ± 6.83
Tb.Sp (μm)	514.93 ± 235.24	437.79 ± 211.86	534.56 ± 222.56	590.16 ± 272.66
SB.Th (μm)	306.57 ± 76.46	251.60 ± 67.43	264.07 ± 28.36	268.20 ± 47.01
Cartilage				
FI (mm)	1.08 ± 0.07	1.22 ± 0.14	1.10 ± 0.16	1.10 ± 0.05
Cg.Th (μm)	493.73 ± 80.73	620.12 ± 114.82^c^	473.82 ± 87.57^b^	571.12 ± 96.73
nCg.Th (μm)	338.64 ± 65.03^b^	465.23 ± 88.92^a,c^	322.19 ± 82.98^b^	409.79 ± 82.56
cCg.Th (μm)	155.09 ± 24.94	167.75 ± 43.01	151.69 ± 28.05	161.33 ± 42.09

Comparison of the histological parameters obtained from undecalcified samples between experimental groups. All variables were normally distributed, except FI. Statistical significance *p* < 0.05: ^a^ vs. CTRL; ^b^ vs. OA; ^c^ vs. CTRL + DC; ^d^ vs. OA + DC

**Figure 3:**
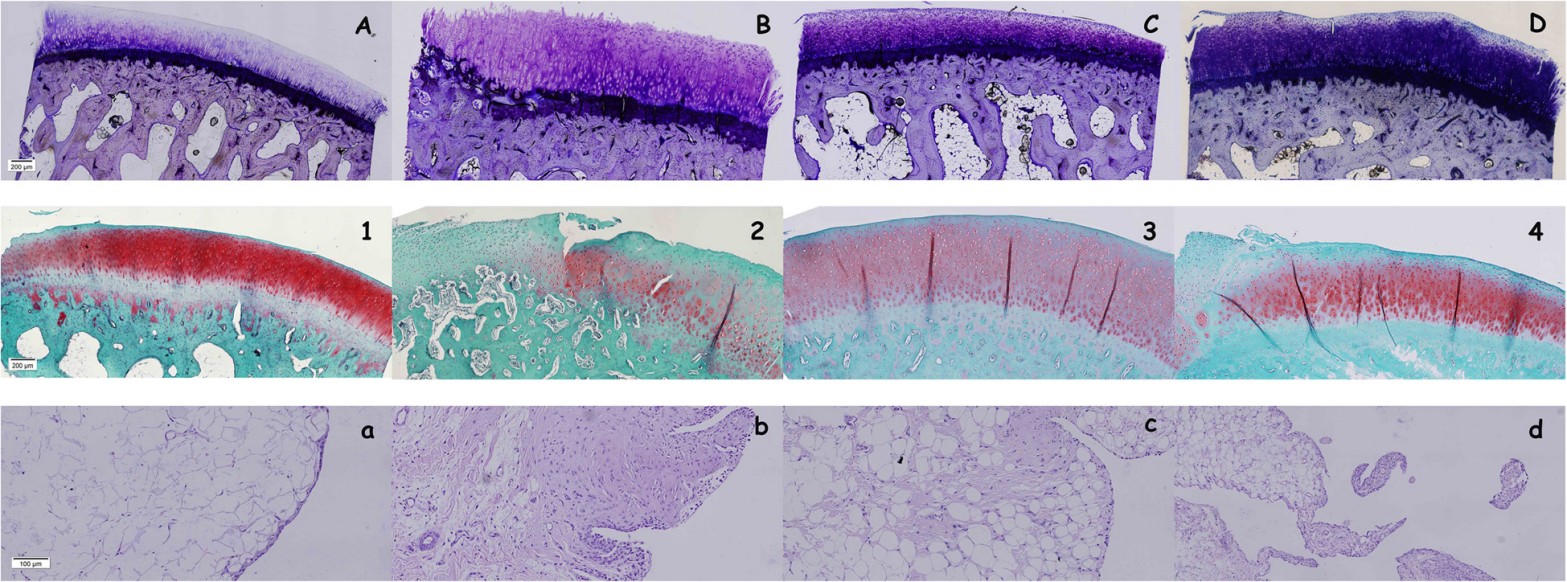
Representative histology images. **A-D**: Lévai-Laczkó stained undecalcified sections (A CTRL; B OA; C CTRL + DC; D OA + DC), magnification 10x. **1**–**4**: safranin-O/fast green stained decalcified sections (1 CTRL; 2 OA; 3 CTRL + DC; 4 OA + DC), magnification 10x. **a-d**: synovial membrane sections stained with H-E (a CTRL; b OA; c CTRL + DC; d OA + DC), magnification 20x

The subchondral bone parameters measured in these calcified slices obtained from CTRL and OA groups were similar and showed no changes in both treated groups (CTRL + DC and OA + DC). Regarding the cartilage-related parameters, non-calcified cartilage thickness (nCg.Th) showed a significant increase in the OA group compared to CTRL (*p* = 0.024) and CTRL + DC (*p* = 0.009). The OA + DC group (operated knee of the diacerein-treated animals) showed a decrease of the nCg.Th with respect to the OA, reaching intermediate values halfway between CTRL/CTRL + DC and OA but showing no significant differences compared to none of them. For the total cartilage thickness (Cg.Th) differences between CTRL and OA groups are not significant, unlike between OA and CTRL + DC groups (*p* = 0.029). In this case, as well as in the non-calcified cartilage, the OA + DC group showed intermediate values between controls and OA. In relation to the cartilage surface undulations (FI) there were no statistical differences between groups, but an almost significant reduction (*p* = 0.06) of fibrillation was found between OA and OA + DC groups (Fig. 3), the latter reaching values similar to both control groups (CTRL and CTRL + DC).

In the microscopic images of the calcified samples stained with Levai-Lazckó (Fig. 3 A-D), despite small differences in the statistical analysis, several differences between groups were observed. Cartilage thickness (Cg.Th) of the OA groups (3B) was thicker than the others, the OA + DC group (3D) reaching an intermediate thickness halfway between controls (3A and 3C) and OA. Another important finding that may be visualised in the images is that the OA group had more superficial fibrillation than the others and the OA + DC surface is similar to those of the controls. The distribution of chondrocytes in the cartilage layer, although the cell distribution and cluster formation was not evaluated in these calcified samples, was different too, showing more disorganisation in the OA group (3B) than in the others. Finally, in the image of the OA group the loss of the part of the calcified cartilage (cCg.Th) could be observed, unlike in the others (3A, 3C, 3D).

### Qualitative histological results

**Figure 4:**
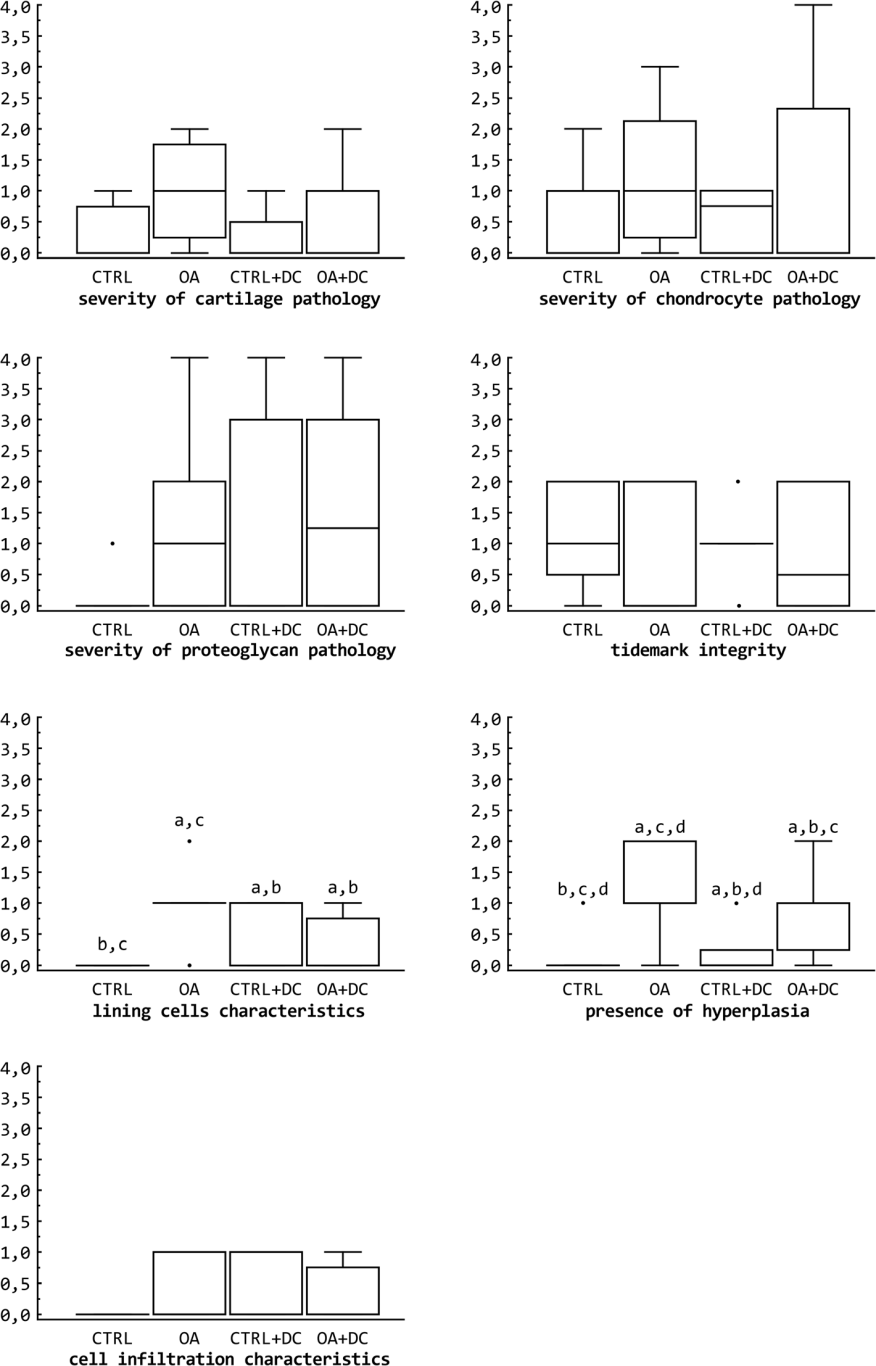
Box plot comparing the scores obtained by the different experimental groups for qualitative microscopic grading of cartilage and synovial alterations (decalcified samples). Outside values were defined as the values that were smaller than the lower quartile minus 1.5 times the interquartile range or larger than the upper quartile plus 1.5 times the interquartile range (inner fences). CTRL: healthy + placebo; OA: osteoarthritis + placebo; CTRL + DC: healthy + diacerein; OA + DC: osteoarthritis + diacerein. All parameters showed a non-normal distribution. Statistical significance p < 0.05: a vs. CTRL; b vs. OA; c vs. CTRL + DC; d vs. OA + DC

The values of microscopic grading for the decalcified cartilage samples and for the synovial membrane are shown in Table 2 and Fig. 4.

**Table 2:** Qualitative histological results

	CTRL	OA	CTRL + DC	OA + DC
	median (25, 75)	median (25, 75)	median (25, 75)	median (25, 75)
	μ ± std.dev	μ ± std.dev	μ ± std.dev	μ ± std.dev
Microscopic grading of cartilage alterations				
Severity of cartilage pathology	0 (0, 0.75)	1 (0.25, 1.75)	0 (0, 0.5)	0 (0, 1)
	0.29 ± 0.49	1 ± 0.82	0.25 ± 0.46	0.5 ± 0.76
Severity of chondrocyte pathology	0 (0, 1)	1 (0.25, 2.5)	1 (0, 1)	1 (1, 3)
	0.57 ± 0.79	1.21 ± 1.15	0.56 ± 0.5	1.81 ± 1.19
Severity of proteoglycan pathology	0 (0, 0)	1 (0, 2)	0 (0, 3)	1.5 (0, 3)
	0.33 ± 0.82	1.29 ± 1.5	1.25 ± 1.83	1.56 ± 1.68
Tidemark integrity	1 (0.5, 2)	0 (0, 2)	1 (1, 1)	0.5 (0, 2)
	1.13 ± 0.83	0.67 ± 1.03	1 ± 0.53	0.88 ± 0.99
Microscopic grading of synovial changes				
Lining cells characteristics	0 (0, 0)^b,c,d^	1 (1, 1)^a,c,d^	0 (0, 1)^a,b^	0 (0, 0.75)^a,b^
	0 ± 0	1 ± 0.63	0.4 ± 0.55	0.29 ± 0.49
Presence of hyperplasia	0 (0, 0)^b,c,d^	1 (1, 2)^a,c,d^	0 (0, 0.25)^a,b,d^	1 (0.25, 1)^a,b,c^
	0.13 ± 0.35	1.17 ± 0.75	0.2 ± 0.45	0.86 ± 0.69
Cell infiltration characteristics	0 (0, 0)	1 (0, 1)	0 (0, 1)	0 (0, 0.75)
	0 ± 0	0.67 ± 0.52	0.4 ± 0.55	0.29 ± 0.49

Comparison between experimental groups with different scores for microscopic grading of cartilage and synovial alterations, obtained from decalcified samples. All parameters showed a non-normal distribution. Statistical significance *p* < 0.05: ^a^ vs. CTRL; ^b^ vs. OA; ^c^ vs. CTRL + DC; ^d^ vs. OA + DC

The parameters that assess changes in non-calcified cartilage showed no differences between the four groups, although the results of the severity of cartilage pathology of the OA + DC group (osteoarthritic knees of the diacerein-treated animals) are halfway between both control groups (CTRL and CTRL + DC) and OA, without any statistical differences between them.

Two of the variables determined in the synovial membrane, that is, the features of the lining cells and the presence of hyperplasia, showed statistical significance between the OA and CTRL group (*p* < 0.05), but there was no presence of inflammatory infiltrates. Between the two diacerein groups (CTRL + DC and OA + DC) the only statistical difference is the presence of hyperplasia. OA + DC shows significant differences compared to OA in both lining cells characteristics and hyperplasia (Fig. 3) but not compared to the controls (CTRL and CTRL + DC). The OA + DC group reaches intermediate values halfaway between OA and CTRL for the three parameters evaluated in the synovial membrane.

As shown by the results of the histologic qualitative assessment of the decalcified samples, diacerein treatment resulted in less alteration of the cartilage surface but not in amelioration of the cellular alterations or in the loss of proteoglycan staining with respect to the osteoarthritic samples of the placebo-treated group (OA). Fig. 3.2 (OA) and 3.4 (OA + DC) shows the chondrocyte disorganisation, with cells not arranged in rows, unlike in the controls (CTRL (3.1) and CTRL + DC (3.3)), and the evident formation of clusters, as well as the loss of the red stain, especially in the superficial layers of the cartilage.

In the synovial membrane of both control groups (Fig. 3.a and 3.c) the lining surface was thin, with one or two layers of cells, the surface was smooth, with no presence of short villi or finger-like hyperplasia, or of inflammatory infiltrations. The OA group (Fig. 3.b) was in the opposite situation, with a thick lining surface, profuse hyperplasia, presence of diffuse inflammatory infiltration and a deposition of fibrous tissue below the intima tissue. Finally, in the OA + DC group (Fig. 3.d), the number of superficial layers of cells tend to approach to the controls, as well as hyperplasia, these samples exhibiting less fibrous subintima tissue than the OA group.

### Micro-CT results

The mean and SD values for the micro-CT parameters are shown in Table 3.

**Table 3:** Micro-CT results

	CTRL	OA	CTRL + DC	OA + DC
	mean ± std.dev	mean ± std.dev	mean ± std.dev	mean ± std.dev
Subchondral bone microstructure				
BV/TV (%)	48.46 ± 7.74^c^	49.37 ± 4.38	56.34 ± 2.96^a^	51.92 ± 4.99
Tb.Th (μm)	265.21 ± 32.62	288.75 ± 60.43	283.20 ± 34.22	281.18 ± 56.18
Tb.Sp (μm)	467.24 ± 122.46^c^	423.12 ± 66.21	354.74 ± 36.25^a^	433.27 ± 54.03
Tb.N (mm^−1^)	1.83 ± 0.20	1.76 ± 0.29	2.01 ± 0.23	1.89 ± 0.30
Tb.Pf (mm^−1^)	−3.27 ± 1.49	−3.24 ± 0.51	−4.16 ± 1.54	−4.01 ± 1.15
SMI	−0.66 ± 0.47	−0.72 ± 0.39	−1.07 ± 0.48	−0.96 ± 0.60
DA	0.71 ± 0.06^c,d^	0.69 ± 0.06^c,d^	0.57 ± 0.09^a,b^	0.57 ± 0.06^a,b^
Volumetric subchondral bone mineral density				
vBMD (mg/cm^3^)	505.90 ± 98.53	512.78 ± 52.52^c^	608.36 ± 24.43^b^	548.69 ± 58.95
Cartilage morphology				
nCg.Th (μm)	309.06 ± 62.99^b^	494.80 ± 169.59^a,c,d^	269.01 ± 39.93^b^	359.75 ± 48.62^b^
nCg.V (mm^3^)	3.37 ± 0.78^b^	5.69 ± 2.72^a,c^	2.81 ± 0.52^b^	3.71 ± 0.50

Mean values ± standard deviation of the micro-CT parameters obtained for all experimental groups. All variables showed a normal distribution, and only vBMD showed no homogeneity of variances. Statistical significance *p* < 0.05: ^a^ vs. CTRL; ^b^ vs. OA; ^c^ vs. CTRL + DC; ^d^ vs. OA + DC

**Figure 5:**
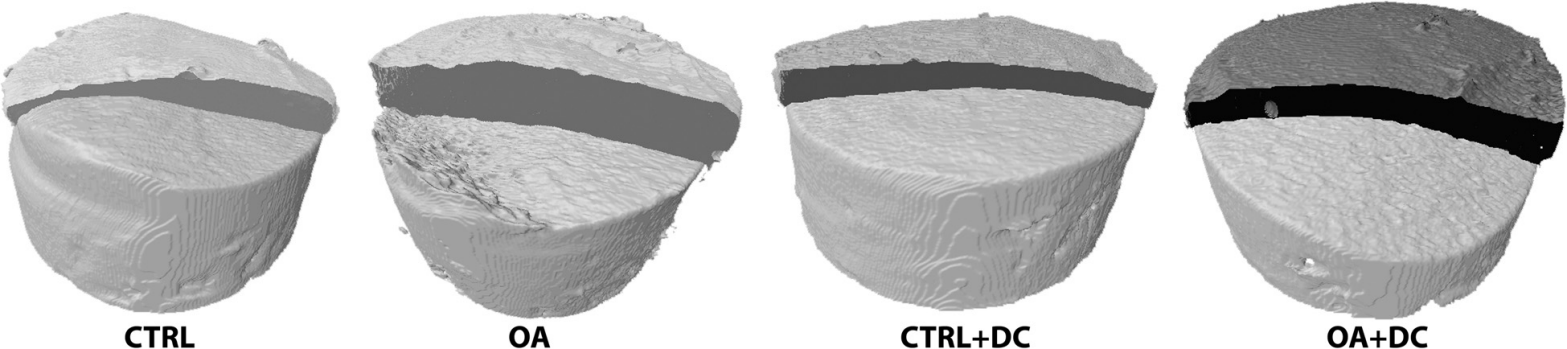
Micro-CT 3D models of a representative sample of each experimental group. Cartilage swelling characteristic of the first stages of OA can be clearly appreciated in OA samples, whereas diacerein-treated samples recover normal cartilage morphology like CTRL

Comparing micro-CT data from the OA group versus the CTRL group, there were no differences in any of the microstructural parameters referring to the subchondral bone or volumetric bone mineral density (vBMD). In contrast, both the thickness of cartilage (nCg.Th) and its volume (nCg.V) are significantly increased in the OA group with respect to CTRL (*p* = 0.003 and 0.018, respectively) and to CTRL + DC (*p* < 0.001 and *p =* 0.003) (Fig. 5).

For the OA + DC group, the subchondral bone measurements show differences only with OA regarding the degree of anisotropy (*p* = 0.004), the rest of the results being similar to those obtained from the other groups. Nonetheless for cartilage parameters, OA + DC showed nCg.V and nCg.Th values close to those of the healthy control group (CTRL); thus, the value of nCg.Th in the OA + DC group presents statistically significant differences compared to OA (*p* = 0.041), whereas nCg.V is almost significant (*p* = 0.053) (Fig. 5).

In the healthy group treated with diacerein (CTRL + DC), the drug showed an anabolic effect on the subchondral bone structure, with an increase in bone volume fraction (BV/TV) versus CTRL (*p* = 0.029), besides a decrease in trabecular separation and degree of anisotropy (*p* = 0.032 and 0.001 respectively). The microstructural parameters measured in cartilage showed no difference between both control groups.

## Discussion

Diacerein has been subject of study regarding its use in the treatment of human OA for some time now. Animal models have been widely used to study the efficacy of therapies in order to improve, fight or prevent OA. Surgically-induced OA models, resulting in joint instability, produce a gradual progression of the joint degeneration and mimic the pathogenesis and pathology of the human traumatic OA [30, 31]. Since the first model carried out by Paatsama (1952) [32], many studies have surgically induced OA using meniscectomy and/or transection of the collateral and cruciate ligaments in different animal species [33]. Partial medial meniscectomy in rabbits results in mild to moderate changes in the joint, resembling those in humans, thus it has been extensively used for testing potential chondroprotective agents [34]. Our animal model was a combination of meniscectomy and ligament transection to avoid the great capacity of rabbits to heal the transected meniscus with fibrous tissue and, similarly to the results obtained by other authors, the results in the present study have shown that this model produced degenerative changes with respect to the healthy contralateral joint [30]. Although the animals were sacrificed at 11 weeks post-surgery, OA seemed to have begun developing, as proven by the swelling of the cartilage. The increase in volume and thickness of cartilage of the OA samples is due to a swelling phenomenon characteristic to the early stages of the disease. Several studies on animals of early OA with magnetic resonance imaging and histomorphometry identified an initial phase of cartilage hypertrophy prior to its degeneration and loss [35, 36]. The explanation has not been found yet, and it is not known whether it is the tissue expression to an inflammatory and repair phenomenon or if it represents a permanent damage of the joint tissue.

Special attention should be paid to the methodology used in this study. Samples of subchondral bone and articular cartilage were analysed using different quantitative and qualitative microscopic methods and micro-CT. Histologic assessment of OA is currently considered as the gold-standard for determining the presence, extent and severity of the disease using Mankin [37] or modified Mankin scoring systems, but recently the histomorphometry using computer analysis systems [24] has been introduced in OA studies with a greater degree of objectivity and reproducibility compared to previous qualitative studies.

Micro-CT has become in recent years the gold-standard for three-dimensional analysis of bone microstructure and its objectivity and reproducibility was comparable to histomorphometry. Although normally when the cartilage structure was quantified using micro-CT, the process involved complex staining techniques with radiopaque agents [38–40], in the present study the scan conditions have been adjusted to achieve a correct cartilage visualisation without contrast agents and it was enough to be able to quantify its morphology.

Our results revealed the potential anti-inflammatory effects of diacerein on osteoarthritis in two different ways: an improvement in the synovial membrane and a reduction in the thickness of the non-calcified cartilage. Previous studies revealed the beneficial effect of this drug not only for cartilage protection, but also for synovitis and reduction in bone erosion in a mice model of inflammatory arthritis [41]. The mechanisms of the anti-inflammatory effect of diacerein have not yet been completely clarified but Tamura *et al.* (2002) [42] demonstrated, using inflammatory animal models, a different spectrum of anti-inflammatory activity of diacerein than those of NSAIDs, which may be due to the inhibition of IL-1 and the production of reactive oxygen species [42, 43]. The histologic evaluation of the synovial membrane in the OA group revealed moderate to severe inflammatory changes with thickening of the lining cell layer, hyperplasia and infiltration with inflammatory cells. In the diacerein-treated group (OA + DC) these changes are milder than in the OA group, although, as in a canine cruciate-deficiency model [12] the results are not statistically significant, as the animals of this OA + DC group obtained values halfway between healthy and osteoarthritic ones. The same results were observed in the cartilage, where a reduction of the thickness is observed in treated animals with respect to the OA group.

In the paraffin-embedded samples there were no differences between groups (although the cartilage pathology is almost significant). A possible explanation could be the substantial variability due to the use of a qualitative subjective scoring system for their evaluation; another explanation (comparing these results with the calcified samples) could be that due to decalcification the integrity of the samples (chiefly bone and calcified cartilage) may have been altered [24]. However, in decalcified samples, the results for the OA + DC group were closer to normality than those obtained for the OA group.

In the samples evaluated by a histomorphometric quantitative methodology, results showed improvement in the diacerein-treated group compared to the one treated with placebo, although differences were not significant. In cartilage parameters there were statistical differences in Cg.Th and nCg.Th between the OA group and the CTRL + DC group and for the latter also with the CTRL group. There were no differences between OA and OA + DC, but nor between OA + DC and CTRL + DC and the tendency of values was to approximate joint values to normal. Regarding FI -an important parameter because it makes a clear distinction between ill and healthy animals [44]- there were no statistical differences between groups, but there is a tendency of approximating values of OA + DC to those of controls.

The results obtained from the undecalcified samples were better than those from the decalcified ones because of a better conservation of the tissue structure and because the computer evaluation has a greater degree of objectivity, accuracy and reproducibility, as well as the micro-CT [24], with comparable results between both. In our study, the subchondral bone showed no statistical differences, so further studies may be needed in this regard.

Micro-CT data have shown an anabolic effect of diacerein on subchondral trabecular microstructure in both healthy and osteoarthritic samples (though less pronounced in the latter). At the same time, the treatment has been able to stop the swelling of cartilage in OA samples, recovering nCg.Th and nCg.V values very close to the CTRL group.

The results of the present animal study conducted in rabbits suggest the possible efficacy of diacerein to slow the progression of the disease in an early model of OA, as shown in previous studies *in vitro* [45], in different animal models [12, 42, 45, 46] and in clinical trials [7, 16, 47]; although the comparison of different studies is difficult because of their high degree of heterogeneity (different models conducted in different animal species and humans, different time schedule and others). To complete this study and correctly evaluate the obtained results, it may be necessary to evaluate the effects of diacerein treatment in the same animal model with a more advanced OA.

## Conclusions

Oral diacerein as OA treatment has been able to reduce cartilage swelling, surface alterations and the inflammation of the synovial membrane in the early stages of OA in a rabbit surgical model. Regarding the subchondral bone, diacerein has shown in healthy bone, but not in the osteoarthritic one, the ability to increase bone volume and mineral density, indicating a surprising effect of this drug on the bone structure, whose results have not been published up to now.

The study also demonstrates the validity of the animal model, and that micro-CT could be a valid technique to detect morphological changes in articular cartilage with comparable results to histomorphometry.

Further studies on long-term OA animal models and well-conducted clinical trials may be required to confirm or exclude the efficacy of diacerein in the treatment of knee OA.

## Abbreviations

OA: Osteoarthritis
SYSADOAs: symptomatic slow-acting drugs for osteoarthritis
NSAIDS: non-steroidal anti-inflammatory drugs
ARRIVE: Animal Research: Reporting *in vivo* experiments
ACLT: anterior cruciate ligament transection
SO: safranin-O staining
H-E: Hematoxylin-eosin
